# Interventions to prevent mother-to-child transmission in breastfeeding mothers with HIV: a systematic review and meta-analysis of randomized controlled trials

**DOI:** 10.1590/S1678-9946202466045

**Published:** 2024-07-29

**Authors:** Fangping Xu, Ying Xiong, Min Gu, Lingling Wan, Yun Wang

**Affiliations:** 1 Jiangxi Maternal and Child Health Hospital, Obstetrical Department, Jiangxi, Nanchang, China

**Keywords:** HIV, Prevention, Mother-to-child transmission, Breastfeeding, Systematic review, Meta-analysis

## Abstract

This study aimed to systematically review interventions to prevent mother-to-child transmission of HIV during breastfeeding. We conducted a systematic review and meta-analysis using specific criteria to identify randomized controlled trials that focused on pregnant and breastfeeding women living with HIV and their children from birth to 2 years of age. We extensively searched electronic databases, including Web of Science, Scopus, PubMed, MEDLINE, EMBASE, Cochrane Central Register of Controlled Trials, and Google Scholar up to October 24, 2023. After screening 3,110 titles and abstracts, we reviewed 306 full texts. Of these, we assessed the quality and risk of bias of fifty-five articles, ultimately identifying seven studies. Four of these studies, which focused on antiretroviral therapy (ART), were included in the meta-analysis. There was little heterogeneity in study methodology and pooled estimates. The postnatal HIV transmission rate was found to be 0.01 (95%CI: 0.00 – 0.02). Therefore, the risk of mother-to-child transmission among breastfeeding mothers with HIV was significantly lower in the intervention groups than in the placebo groups. Analysis of funnel plots and Egger’s test (p = 0.589) showed no evidence of publication bias. In addition to the four articles, two studies compared different ART regimens and one study compared the administration of high-dose vitamin A to the mother or the child. The results suggest that the use of ART significantly reduces the risk of postnatal HIV transmission compared with placebo. However, the effectiveness of different ART regimens or other therapies, including high-dose vitamin A, is unclear.

## INTRODUCTION

The human immunodeficiency virus (HIV) weakens the immune system, making the body susceptible to opportunistic infections^1^. While unprotected sex is the primary mode of HIV transmission, a significant number of cases also result from mother-to-child transmission (MTCT)^2^. MTCT is a major contributor to the HIV epidemic, accounting for approximately 9% of new infections worldwide^3^. Most HIV infections in children occur during pregnancy, childbirth, or breastfeeding^4,5^.

Prevention of Mother-to-Child Transmission (PMTCT) services are estimated to have prevented HIV infection in around 1.4 million children from 2010 to 2014, one of the most significant public health achievements of the past two decades^6^. The PMTCT strategy involves four core elements: universal maternal testing and prenatal counseling, use of antiretroviral therapy (ART) during pregnancy and delivery, postnatal administration of ART to the infant, and avoidance of breastfeeding. In addition, Cesarean section is recommended for mothers with a high plasma viral load or if viral load is undetectable. This comprehensive set of preventive interventions can reduce the risk of fatal infection to less than 1%^7,8^. Infants born to women living with HIV have a 15–30% chance of acquiring the virus during pregnancy or delivery and a 5–36.4% chance of acquiring it during breastfeeding if left untreated^9,10^.

The transmission of HIV during breastfeeding was first recognized in 1985, sparking ongoing debate and policy discussions on the prevention of mother-to-child transmission^11^. Breastfeeding, especially exclusive breastfeeding, is widely recognized for its well-documented benefits over infant formula feeding, especially in resource-limited settings^12^. As a result, the World Health Organization (WHO) recommends exclusive breastfeeding for infants for the first six months, regardless of the mother’s HIV status^11^. However, due to the increased risk of transmission associated with prolonged exposure to HIV-infected breast milk, interventions to reduce the duration of such exposure should be considered. While encouraging exclusive breastfeeding is an important part of prevention strategies, the combination of breast milk and complementary foods for infants under 6 months of age is associated with an increased risk of transmission^13^.

The risk of transmission via breast milk is influenced by maternal factors such as higher viral load in peripheral blood and breast milk. To mitigate this, the use of antiretroviral drugs by breastfeeding mothers is considered an intervention to reduce transmission to the infant^14,15^. Furthermore, for infants who have been breastfed for months, strengthening the immune system against HIV infection using prophylaxis can also reduce the risk of transmission^16^. Supportive counseling interventions during breastfeeding can also be effective. Every mother should receive information on alternative feeding strategies for her infant in critical situations, with ongoing support throughout the breastfeeding period. Appropriate counseling is essential to identify the right time for weaning, while ensuring access to appropriate complementary foods to prevent malnutrition^17,18^. There is a critical need for counseling among lactating women, as the rate of virus recurrence is high. Even mothers with suppressed viral loads at the time of delivery should receive the necessary care until the end of breastfeeding^14^.

Manji *et al*.^19^ evaluated the role of breastfeeding in mother-to-child transmission (MTCT) and found that each additional month of breastfeeding resulted in an 18% reduction in transmission. Another study found that providing infants with an oral dose of nevirapine (NVP) for six months or longer was effective in reducing HIV transmission during breastfeeding^20^. In addition, a study was conducted in Tanzania during a period when antiretroviral drugs (ARVs) were not available. This study showed that breastfeeding provided protection against HIV infection and death, particularly during the first five months of life^21^. Bansaccal *et al*.^22^ also showed that many preventive measures, including the use of antiretroviral drugs for mothers and infants, along with close follow-up, effectively prevented HIV infection in infants. Puchalski Ritchie *et al*.^23^ conducted a systematic review and meta-analysis and concluded that current evidence does not support the effectiveness of interventions aimed at improving maternal and infant uptake and retention in PMTCT care. However, their findings indicate a potential benefit of integrating HIV and antenatal care, which may enhance ART use during pregnancy. The systematic review conducted by Bispo *et al.* ^24^ found evidence of a significant reduction in postnatal HIV transmission risk with maternal ART coverage. However, the risk of transmission increased once PMTCT ART was discontinued after six months, supporting the current World Health Organization recommendations for lifelong ART for all.

Despite progress in reducing mother-to-child transmission (MTCT) of HIV during breastfeeding, significant challenges remain, as breastfeeding continues to be a major contributor to new infections in children, posing a substantial obstacle to the goal of eliminating MTCT of HIV by 2030^14^. Further research in this field is needed to address these challenges. Systematic reviews and meta-analyses play a crucial role in evaluating the effectiveness of different breastfeeding interventions in preventing mother-to-child transmission of HIV. These studies help to identify successful strategies and provide evidence-based guidelines for health care providers and policy makers. They also highlight gaps in current interventions and areas for improvement. By combining findings from multiple studies, researchers can comprehensively understand how to effectively prevent mother-to-child transmission of HIV during breastfeeding. The results of such studies will contribute to the global effort to eliminate mother-to-child transmission of HIV. Therefore, this systematic review and meta-analysis aimed to investigate the impact of breastfeeding interventions on the prevention of mother-to-child transmission of HIV.

## MATERIALS AND METHODS

### Eligibility criteria

In this systematic review and meta-analysis, specific inclusion and exclusion criteria were used to identify relevant studies and analyze their findings. The review focused on randomized controlled trials involving pregnant and breastfeeding women living with HIV (without co-infections such as Herpes Simplex Virus) and their children from birth to two years of age. These studies investigated various interventions and practices, such as antiretroviral therapy and feeding modalities, aimed at preventing mother-to-child or vertical transmission of HIV during breastfeeding. Original articles, proceedings, early access publications, and articles in press, including short surveys, were included. The language criterion was restricted to English, and articles published before 2000 were excluded due to potential changes in health care systems. Older data may not provide an up-to-date perspective, especially considering the introduction of the triple combination ART regimen in public health programs in 2004.

### Information sources

Electronic databases, including Web of Science, Scopus, PubMed, MEDLINE, EMBASE, Cochrane Central Register of Controlled Trials, and Google Scholar^25^, were extensively searched up to October 24, 2023. Furthermore, the search was extended by reviewing the reference lists of the studies included in the analysis to ensure a comprehensive investigation of the relevant literature.

### Search strategy

A search strategy was carefully developed in accordance with the peer-reviewed guideline on electronic search strategies^2^. This approach was tailored to each database search engine to ensure the highest quality of results. Detailed search strategies are provided in Supplementary File S1.

### Selection process

**Figure 1:**
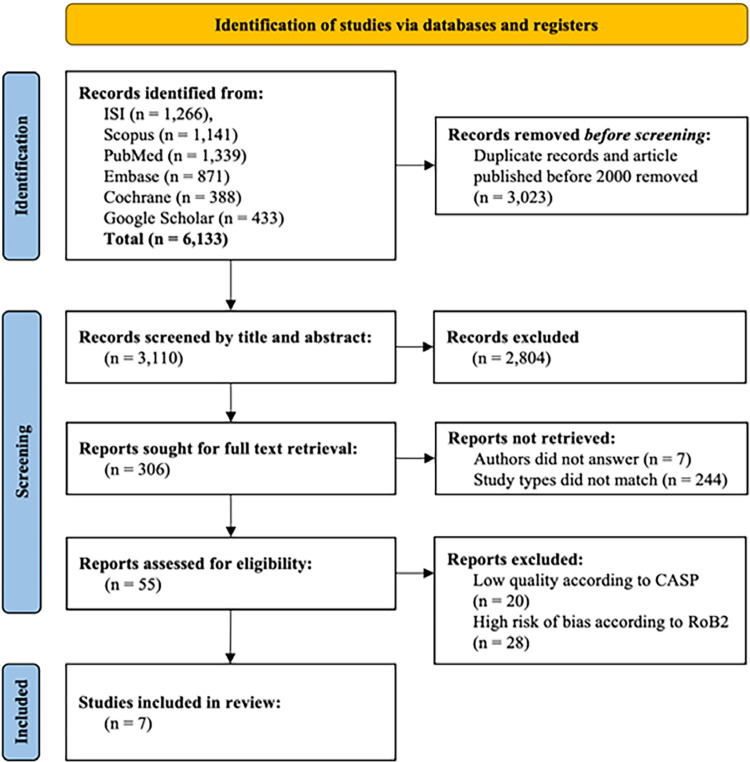
Flowchart of the screening process.

As a first step, all search results from the databases were imported into Google Sheets and duplicates were removed using the ‘Remove duplicates’ function. Subsequently, two researchers independently screened articles based on eligibility criteria derived from the titles and abstracts. Any disagreements between the two researchers in the selection of studies were resolved by full-text review with the involvement of a third researcher. In addition, the corresponding authors of eligible studies were contacted via ResearchGate. However, although nine authors were contacted, only two responded within two weeks. The screening process was illustrated using the Preferred Reporting Items for Systematic Reviews and Meta-Analyses (PRISMA) 2020 flow diagram^3^ (Figure 1).

### Data collection process

A data extraction form was developed to ensure consistency, comparing the completed forms for the first three articles using a calibration exercise. Subsequently, two authors independently conducted data extraction for all articles. Any discrepancies were resolved via consensus discussions with a third author. The data extraction form included information such as country, objectives, inclusion/exclusion criteria, study population, intervention and comparison/control groups, feeding methods and duration, transmission rates, adverse events, infant/mother mortality, mode of delivery, follow-up details, risk ratio (95% confidence interval), and adjusted statistics. The primary outcome was the vertical transmission rate (percentage), while the secondary outcomes focused on the effectiveness of breastfeeding and/or complementary feeding mode (percentage) and duration (mean) in preventing vertical transmission.

### Assessment of risk of bias

To assess the quality of the studies, the CASP Randomized Controlled Trial Standard Checklist was used, which consists of 11 questions divided into four sections: A) validity of the basic study design; B) soundness of the study methodology; C) reporting of the results; and D) implications of the results at the local level^26^. Studies that did not meet more than half of the checklist items or were unclear were excluded. In addition, a duplicate assessment of the risk of bias for each study was conducted using the Revised Cochrane risk-of-bias tool for randomized trials (RoB 2). This tool evaluates potential sources of bias in five domains: (1) bias arising from the randomization process; (2) bias due to deviations from the intended interventions; (3) bias due to missing outcome data; (4) bias in the measurement of the outcome; and (5) bias in the selection of the reported outcome^27^. Studies with concerning factors or that were at high risk in two or more domains were excluded. Seven articles were of low risk and high quality.

### Data synthesis and analysis

A descriptive synthesis of study results was conducted for all studies and reported in both narrative and tabular form. HIV transmission rates with confidence intervals and pooled estimates were used in the synthesis and presentation of results. To estimate the intervention effects, random effects meta-analysis with a heterogeneity score was performed using STATA 16 (StataCorp LLC, College Station, TX, USA). The random effects model was chosen because of methodological differences. Heterogeneity was assessed using the I^2^ statistic, with I^2^ ≥ 50% indicating significant heterogeneity.

### Publication bias

Publication bias was assessed using funnel plots and Egger’s test. Funnel plots provide a visual representation of the effect estimates of studies plotted against their standard errors. In the absence of publication bias, the funnel plot should have a symmetrical inverted funnel shape. The funnel plots were visually inspected for signs of asymmetry, and Egger’s test was used to quantitatively evaluate the presence of publication bias. A p-value below 0.10 was considered significant publication bias. If publication bias was identified, the plan was to employ trim-and-fill analysis to account for its influence on the overall effect estimate.

## RESULTS

### Literature search

Of the initial 6,133 articles identified by the database search, 3,023 duplicates were removed. The remaining 3,110 titles and abstracts were screened, and 306 full-text articles were reviewed. A total of 55 articles were then assessed for quality and risk of bias, resulting in the inclusion of seven articles in the study (Figure 1).

### Study characteristics

The studies included in this review were carried out in various African countries, including Kenya, Burkina Faso, South Africa, Uganda, Zambia, Tanzania, Zimbabwe, and Ivory Coast, from 2002 to 2016. The number of mothers and infants included in these studies ranged from 148 to 4,495. The age of the participating mothers ranged from 22 to 31.1 years. Follow-up visits were conducted at various time points, ranging from prenatal visits at 34 weeks of gestation to 50 weeks postpartum. Of the included studies, five had two groups for intervention and placebo^7,8,28-30^, one had two experimental groups, and another had three experimental groups with one placebo group^11,12^.

### Interventions to prevent mother-to-child transmission in breastfeeding mothers with HIV

The seven articles included in this review used different interventions. In three of the articles, zidovudine regimens with or without other antiretroviral therapy were administered^7,12,30^. In one article, mothers received zidovudine prophylaxis (300 mg orally twice a day) from 36 weeks of gestation to delivery. At the onset of labor, a single additional dose of ZDV (300 mg every 3 h until delivery) was administered. After delivery, no antiretroviral medication was given to either the baby or the mother^30^. In another study, long, intermediate, and short zidovudine plus lamivudine regimens were administered^12^. In another, all mothers received zidovudine from 28 weeks of pregnancy until birth, a single intrapartum dose of nevirapine, and then zidovudine-lamivudine for 7 days postpartum. In addition, all infants received nevirapine for 7 days from birth. Infants also received lopinavir and ritonavir doses based on their weight, or lamivudine doses if certain weight criteria were met. This study did not include a placebo group^7^. Two other interventions involved the administration of nevirapine with increasing doses as the infant aged (extended daily nevirapine)^8,29^. In one article, mothers received valacyclovir (500 mg twice a day) from 38–40 weeks of gestation^28^. Another intervention involved the administration of a single high dose of maternal/neonatal vitamin A (400,000 IU for the mother and 50,000 IU for the infant)(11) (Table 1). One study that did not have a placebo group^7^ and two other studies that had more than two groups^7,11^ were not included in the meta-analysis.

**Table 1:** Characteristics of the included studies.

Article	Country	Objective	Inclusion/ exclusion criteria	Study population		Intervention group	Comparison/ control group	Feeding		Visits	Transmission rate
	
				Participants (n)	Participant characteristics			Mode	Duration		
Drake *et. al* ^.2^8	Kenya	To quantify the effect of HSV-2 suppression on reducing plasma, cervical, and breast milk HIV-1 RNA levels among pregnant and postpartum Kenyan women coinfected with HIV-1 and HSV-2.	**Inclusion criteria:** ≥ 18 years of age, HIV-1 and HSV-2 seropositive, 28–32 weeks of gestation, CD4 count > 0.250 cells/mm^3^. **Exclusion criteria:** hypersensitivity to acyclovir or valacyclovir, clinical indication for HAART (WHO stage 3 or 4).	**148 subjects enrolled** 74 randomized to each arm 146 women were included in analyzes	**Mothers:** n (%/weeks): **Gestational age** (weeks) Valacyclovir = 39 (38–40), Placebo = 39 (38–41) **Infants:** Median (IQR) or n (%): **B** **irth weight (kg)** Valacyclovir = 3.2 (2.9–3.5), Placebo = 3.0 (2.9–3.5) **Female** Valacyclovir = 38 (53), Placebo = 47 (66)	**A) valacyclovir group (n = 74)** valacyclovir (500 mg twice a day) 51 included in pregnancy analyzes 72 included in postpartum analyzes	**B) Placebo group (n = 74)** 49 included in pregnancy analyzes 73 included in postpartum analyzes	Most infants were breastfed	**Median:** 6.0 months in the valacyclovir arm 5.3 months in the placebo arm (IQR, 3.4–6.5, both arms).	**Antenatal visits:** at 34 and 38 weeks of gestation **Postpartum follow-up visits:** at 2, 6, 10, and 14 weeks and 6, 9, and 12 months postpartum.	**HIV-1 transmission rate:** 10 infants (6 valacyclovir, 4 placebo) acquired HIV-1 by 12 months for a transmission rate of 7.0%. There was no difference in transmission between arms (hazard ratio [HR], 1.45; 95% CI, 0.41–5.12). **HIV-1–free survival:** similar between study arms (HR, 0.86; 95% CI, 0.33–2.22).
Nagot *et. al*.7	Burkina Faso, South Africa, Uganda, and Zambia	To compare the effect of lopinavir–ritonavir and lamivudine on the rate of HIV-1 transmission and adverse events in infants exposed to HIV-1 in Africa.	**Inclusion criteria: Infants:** 7 days of age; singleton; breastfed by their mothers at the 7^th^ day of life; negative HIV-1 DNA PCR at the 7^th^ day of life; received PMTCT; **Mothers:** ≥ 18 years of age, intending to continue breastfeeding, infected with HIV-1, not eligible for ART nor taking ART, received perinatal antiretroviral prophylaxis during pregnancy or delivery. **Exclusion criteria: Infants:** clinical signs or biological abnormalities of grade 2 ≤ on DAIDS adverse events grading tables, haemoglobin < 120 g/L, neutrophils < 1200 cells per μL, serious congenital malformations or birthweight ≤ 2.0 kg.	**1,273 infants exposed to HIV-1 enrolled** 1,236 infants included in the modified intention-to-treat analysis	**Mothers:** Median age: 27·1 years (IQR 23·3–31·1). **Infants: Boys** Lopinavir–ritonavir group = 322 (51%) Lamivudine group = 335 (53%) **Girls** Lopinavir–ritonavir group = 309 (49%) Lamivudine group = 301 (47%) **Birthweight (g)** Lopinavir–ritonavir group = 3000 (2740–3350) Lamivudine group = 3000 (2800–3325)	**A) lopinavir–ritonavir (n = 615)** **Mothers:** zidovudine from 28 weeks of pregnancy until birth, intrapartum single-dose nevirapine, zidovudine–lamivudine for 7 days after birth. **Infants:** 7 days of nevirapine from birth. 40 mg of lopinavir and 10 mg of ritonavir twice a day if weighing 2–4 kg, and 80 mg and 20 mg, twice a day if weighing > 4 kg	**B) lamivudine (n = 621)** **Mothers:** zidovudine from 28 weeks of pregnancy until birth, intrapartum single-dose nevirapine, zidovudine–lamivudine for 7 days after birth. **Infants:** 7 days of nevirapine from birth. Lamivudine: 75 mg twice a day if weighing 2–4 kg, 25 mg twice a day if weighing 4–8 kg, and 50 mg twice a day if weighing > 8 kg	**Breastfeeding only**	**Median:** Lopinavir–ritonavir group = 41.1 weeks (IQR 34.4–45.6), Lamivudine group = 41.4 weeks (35.9–46.7).	2 weeks after enrolment, then every 4 weeks until week 50.	**Infant HIV-1 acquisition:** 17 HIV-1 infections were confirmed in infants: lopinavir–ritonavir = 8, 1.4% (95% CI 0.4–2.5) lamivudine = 9, 1.5% (0.7–2.5) cumulative postnatal transmission rate (p = 0.83). Hazard ratio [HR] of lopinavir–ritonavir versus lamivudine of 0.90, 95% CI 0.35–2.34; p = 0.83. Pooling the two groups, the cumulative transmission rate was 0.7% (n = 9; 95% CI 0.2–1.2) at 26 weeks and 1.5% (n = 17; 0.8–2.2) at 50 weeks.
Coovadia *et. al.* ^8^	South Africa, Tanzani a, Uganda, Zimbabwe	To assess the incremental safety and efficacy of extension of prophylaxis after 6 months.	Eligible women and infants were enrolled within 7 days of delivery. **Inclusion criteria: Mothers:** ≥ 18 years of age. **Infants:** negative HIV-1 DNA PCR, birthweight 2000 g ≤. negative HIV-1 DNA PCR based on a specimen obtained within 21 days before randomization, breastfed. **Exclusion criteria:** serious medical disorders that could interfere with study participation, development of HIV-1 infection.	**1,700 infants enrolled** 1,527 infants randomized **1,505 women infected with HIV-1**	**Mothers: Median age** (years) Nevirapine group = 27 (23–30) Placebo group = 27 (23–31) **Infants: Sex (Male)** Nevirapine group = 363 (48%) Placebo group = 398 (52%) **Sex (Female)** Nevirapine group = 395 (52%) Placebo group = 365 (48%) **Median birthweight (g)** Nevirapine group = 3100 (2800–3400) Placebo group = 3100 (2800–3400)	All enrolled infants received open-label nevirapine (10 mg/mL oral suspension) once a day during the first 6 weeks of life. After this treatment (at 6–8 weeks of age): **A) Extended nevirapine group (n = 759)** The nevirapine dose increased with age, ranging from 20 mg once a day at 6–8 weeks of age to 28 mg once a day at 5–6 months of age.	All enrolled infants received open-label nevirapine (10 mg/mL oral suspension) once a day during the first 6 weeks of life. After this treatment (at 6–8 weeks of age): **B) Placebo group (n = 763)**	**Breastfeeding only**	Women were counselled to exclusively breastfeed for 6 months. More than 95% of infants in both groups were no longer breastfed at the 9-month study visit.	Infant study visits took place within 7 days postpartum, at 2, 5, 6, and 8 weeks, and at 3, 6, 9, 12, and 18 months.	**Extended nevirapine group:** 1.1% (95% CI 0.3–1.8) from 6 weeks to 6 months of age. **Placebo group:** 2.4% (1.3–3.6) (1·3% difference, 95% CI 0–2.6; p = 0.049). equating to a 54% reduction in HIV-1 transmission. Study interventions stopped at 6 months of age. Differences between study groups were no longer significant at 9 and 12 months of age.
Fowler *et. al*.^29^	South Africa, Tanzania, Uganda, and Zimbabwe.	To compare the efficacy and safety of infant nevirapine (NVP) among HIV-exposed breastfed infants randomized at 6 weeks to 6 months to receive NVP or placebo for postnatal infection prevention.	**Inclusion criteria:** **Mothers:** women infected with HIV, ≥ 18 years of age, no other serious illnesses **Infants:** not infected with HIV based on a specimen obtained within 7 days of birth, birth weight of ≥ 2000 g, no life-threatening conditions, breastfed. **Exclusion criteria:**	Women and their **infants (n = 1,522)**	**Mothers:** median age of 27 years **Infants:** median birth weight of 3100 g 52% of infants in each study arm were men.	**A) Extended daily NVP (n = 759)** All enrolled infants received open-label NVP (10 mg/mL oral suspension) once a day during the first 6 weeks of life. NVP administered once a day until 6 months of age or until cessation of breastfeeding (whichever came first).	**B) Placebo (n = 763)**	**Breastfeeding only**	Median: 184 days in both study arms; by 12 months, cessation of breastfeeding was reported for 95% of infants.	Infant follow-up study visits were scheduled at 2, 5, 6, and 8 weeks and at 3, 6, 9, 12, and 18 months.	39 postnatal HIV infections from 6 weeks to 18 months. HIV infection rates at 6 weeks and 6 months of age were significantly different between study arms: 1.1% (95% CI 0.3–1.8) in the NVP arm versus 2.4% (95% CI: 1.3% to 3.6%) in the placebo arm, p = 0.049. By 18 months, there were 16 infections in the NVP arm and 23 infections in the placebo arm, with a cumulative postnatal infection rate of 2.2% (95% CI: 1.1–3.3) versus 3.1% (95% CI: 1.9–4.4, p = 0.28), translating into HIV-free rates of 97.8% in the NVP arm versus 96.9% in the placebo arm.
Humphrey *et. al.* ^11^	Zimbabwe	To investigate the effect of single-high-dose maternal/neonatal vitamin A supplementation on MTCT, HIV-free survival, and mortality in infants exposed to HIV.	**Inclusion criteria: Mother-infant pairs:** neither had an acutely life-threatening condition, the infant was a singleton, birth weight ≥ 1500 g. **Exclusion Criteria:**	**14,110 mother-infant pairs 4,495 infants**	**Maternal age, mean ± SD, years:** Aa = 25.6 ± 5.0 Ap = 25.6 ± 4.9 Pa = 25.5 ± 5.0 Pp = 25.6 ± 5.0 **Infant birth weight, mean ± SD, kg:** Aa = 2.88 ± 0.45 Ap = 2.92 ± 0.47 Pa = 2.91 ± 0.48 Pp = 2.94 ± 0.47 **Male sex:** Aa = 534 (48.4) Ap = 593 (52.7) Pa = 570 (49.8) Pp = 585 (52.2) **Gestational age, mean ± SD, weeks:** Aa = 39.1 ± 1.5 Ap = 39.2 ± 1.5 Pa = 39.1 ± 1.6 Pp = 39.1 ± 1.4	**Aa Group (n = 3,529)** HIV+ Mothers (n = 1,103) HIV- Infants (n = 1,012) **Ap Group (n = 3,529)** HIV+ Mothers (n = 1,126) HIV- Infants (n = 1,006) **Pa Group (n = 3,530)** HIV+ Mothers (n = 1,144) HIV- Infants (n = 1,026) A) maternal vitamin A supplementation (400,000 IU), a) infant vitamin A supplementation (50,000 IU), P) maternal placebo, p) infant placebo.	**Pp Group (n = 3522)** HIV+ Mothers (n = 1122) HIV- Infants (n = 1020)	**Breastfeeding only**	All but 4 mothers infected with HIV initiated breastfeeding, and 97%, 92%, 66%, and 19% were still breastfeeding at 6, 12, 18, and 24 months postpartum, respectively. These proportions did not differ between treatment groups (for all x2 tests [3 df], P > 0.7).	Visits took place at 6 weeks, 3 months, and then every 3 months for 12–24 months. 24%, 48%, and 100% of the pairs were reassigned to 24-month, ≥ 18-month, and ≥ 12-month follow-up, respectively.	**Aa Group:** Infection events at 12 and 24 months: 278 (29.6%: CI: 26.6-32.5) and 297 (35.9%, CI: 31.9 – 40.3), respectively **Ap Group:** Infection events at 12 and 24 months: 333 (35.2%%: CI: 32.4 – 38.6) and 346 (42.8%, CI: 36.7 – 51.8), respectively **Pa Group:** Infection events at 12 and 24 months: 332 (35.2%%: CI: 32.1 – 38.2) and 347 (40.5%, CI: 36.3 – 46.0), respectively **Pp Group:** Infection events at 12 and 24 months: 293 (31.9%: CI: 28.6 – 34.6) and 310 (35.3%, CI: 31.8 – 39.2), respectively
Jamieson *et. al* ^.3^0	Ivory Coast	To examine the maternal risk factors for HIV-1 transmission to children aged 1 and 24 months in a population of lactating women infected with HIV in Ivory Coast.	**Inclusion criteria:** pregnant women seropositive for HIV-1 **Exclusion criteria:** women seropositive for HIV-2, previous treatment with ZDV	124 infants in the placebo group 126 infants in the treatment group	**Mothers: Age (years)** ≤ 25: 106 (42.4) > 25: 144 (57.6)	**A) Oral ZDV prophylaxis (300 mg orally twice a day) (n = 126)** treatment from 36 weeks of gestational age until delivery. Upon onset of labor, a single additional dose of ZDV was administered (300 mg every 3 h until delivery). After delivery, antiretroviral medication was not given to either the baby or the mother.	**B) Placebo (n = 124)**	**Breastfeeding only**	All but 2 children were breastfed from birth, and only 2.4% (6/249) were weaned at the 3-month visit.	Before delivery, women were enrolled at 34 weeks and followed up every 2 weeks until delivery. After delivery, women and infants were followed up at 1 and 3 months and then every 3 months until 24 months after delivery.	Of the 250 infants, 42 were detected as infected by the age of 1 month and an additional 20 were detected as infected from 1 to 24 months of age. Two of the weaned children tested positive at the age of 1 month. Cumulative transmission: the overall risk of transmission in the ZDV group was 11.9% and 22.1% by 1 and 24 months, respectively. In the placebo group, the cumulative transmission risk was 21.9% and 29.2% by 1 and 24 months, respectively.
Petra Study Team^12^	South Africa, Uganda, and Tanzania.	To assess the efficacy of short-course regimens with zidovudine and lamivudine in a predominantly breastfeeding population.	**Inclusion criteria:** ≥ 18 years of age; evidence of HIV-1 infection; gestational period < 36 weeks; absence of severe fetal anomalies; absence of life-threatening disease; hemoglobin levels over 8 g/dL at enrolment. **Exclusion criteria:**	**1,457 women** 366 in regimen A (380 infants; 14 twins) 371 in regimen B (382 infants; 11 twins) 368 in regimen C (377 infants; 9 twins) 352 in the placebo regimen (362 infants; 10 twins)	**Mothers: Age (years, median [IQR])** Regimen A: 26 (23–29) Regimen B: 26 (23–30) Regimen C: 26 (23–30) Placebo: 26 (22–30) **Infants: Male** Regimen A: 199 (53%) Regimen B: 205 (54%) Regimen C: 185 (50%) Placebo: 181 (50%) **Female** Regimen A: 178 (47%) Regimen B: 175 (46%) Regimen C: 187 (50%) Placebo: 177 (50%) **Birthweight (kg, median [range])** Regimen A: 3.1 (2.8–3.4) Regimen B: 3.1 (2.8–3.4) Regimen C: 3.1 (2.8–3·4) Placebo: 3.1 (2.8–3.4)	* Longest drug regimen (Regimen A): (n = 366 infants) From week 36 of pregnancy until the onset of labor: zidovudine (zi) 300 mg plus lamivudine (la) 150 mg twice a day **During labor:** zi 300 mg every 3 h and la 150 mg every 12 h until delivery. **First 7 days postpartum:** women: zi 300 mg plus la 150 mg twice a day newborns: zi 4 mg/kg plus la 2 mg/kg twice a day. *Intermediate regimen (Regimen B): (n = 371 infants) Started at the onset of labor: zi 600 mg and la 150 mg followed by zi 300 mg every 3 h and la 150 mg every 12 h until delivery. **First 7 days postpartum:** women: zi 300 mg plus la 150 mg twice a day newborns: zi 4 mg/kg plus la 2 mg/kg twice a day. * Shortest regimen (Regimen C): (n = 368 infants) women: zi 600 mg and la 150 mg at the onset of labor, then zi 300 mg every 3 h and la 150 mg every 12 h until delivery.	**Placebo: (n = 352 infants)**	**Breastfeeding only**	1081 (74%) of the women breastfed during a median period of 28 weeks (IQR 7–59 weeks). Initiated breastfeeding: Regimen A: 271 (74%) Regimen B: 270 (73%) Regimen C: 273 (76%) Placebo: 267 (74%)	Follow-up visits at weeks 1, 3, and 6, and months 3, 6, 9, 12, 15, and 18.	At week 6, HIV-1 transmission rates were 5.7% for regimen A, 8.9% for regimen B, 14.2% for regimen C, and 15.3% for the placebo group (p = 0.0002). After 18 months, these proportions were 14.9% (95% CI 9.4–22.8), 18.1% (12.1–26.2), 20.0% (12.9–30.1), and 22·2% (15.9–30.2), respectively. Following week 6, continued HIV-1 transmission was rarely seen among non-breastfed children and predominantly occurred in breastfed infants. Due to weaning and HIV transmission, the proportion of breastfed children who were not infected with HIV-1 (the children at risk) declined over time, from 65% after 3 months of follow-up to 53% after 6 months, 31% after 1 year and 20% after 18 months.

### Overall HIV transmission

Of the seven studies, the maximum duration of follow-up varied from 24 to 96 weeks. One article reported the transmission rate at 24 weeks^8^, one at 48 weeks^28^, one at 50 weeks^7^, two at 72 weeks^12,29^, and another two at 96 weeks^11,30^. All infants were exclusively breastfed during the study.

**Figure 2:**
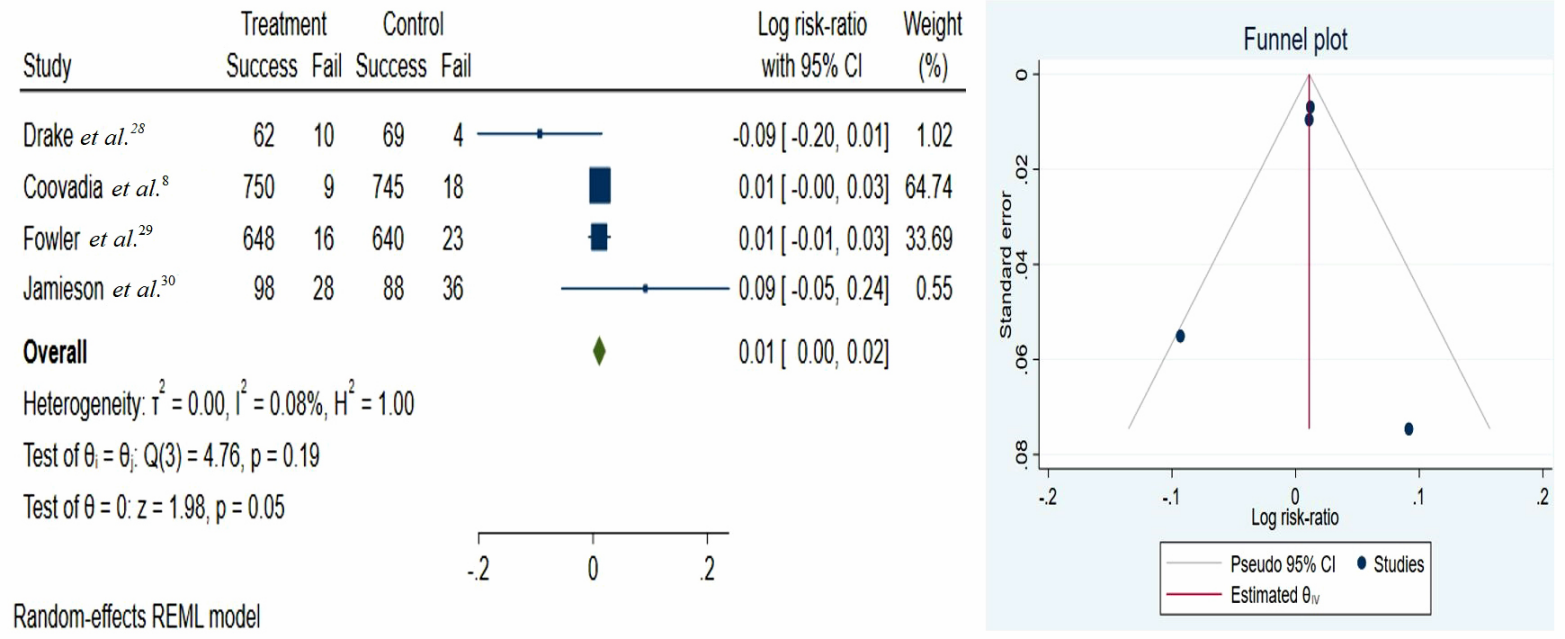
Forest and funnel plots of the risk of mother-to-child transmission in breastfeeding mothers with HIV, with 95% confidence intervals.

According to data from four articles that administered ART (8, 28-30), the overall risk of postnatal HIV transmission rate was 0.01 (95%CI: 0.00 - 0.02). Therefore, the risk of mother-to-child transmission in breastfeeding mothers with HIV was found to be very low in the intervention groups compared with the placebo groups. Heterogeneity analysis showed homogeneity among the studies, with a Tau^2^ of 0.0 and a Chi^2^ of 4.76 (p = 0.19) (Figure 2). According to the funnel plots and Egger’s test (no small study effects, z = −0.54, p = 0.589), there was no evidence of publication bias.

In addition, the study by Nagot *et al.* ^7^, which was not included in the meta-analysis, showed a hazard ratio (HR) of 0.90 for lopinavir–ritonavir versus lamivudine (95% CI: 0.35–2.34; p = 0.83). When the two groups were pooled, the cumulative transmission rate was 0.7% (n = 9; 95% CI: 0.2–1.2) at 26 weeks and 1.5% (n = 17; 0.8–2.2) at 50 weeks. The risk of transmission remained low with both treatments. The results of the study by Humphrey *et al*.^11^ also showed that in the maternal vitamin A supplementation (400,000 IU) and infant vitamin A supplementation (50,000 IU) group, the number of infection events at 12 and 24 months was 278 (29.6% CI: 26.6–32.5) and 297 (35.9%, CI: 31.9–40.3), respectively. In the maternal vitamin A supplementation (400,000 IU) and infant placebo group, the number of infection events at 12 and 24 months was 333 (35.2% CI: 32.4–38.6) and 346 (42.8%, CI: 36.7–51.8), respectively. In the maternal placebo and infant vitamin A supplementation (50,000 IU) group, the number of infection events at 12 and 24 months was 332 (35.2%: CI: 32.1–38.2) and 347 (40.5%, CI: 36.3–46.0), respectively. Lastly, in the maternal and infant placebo groups, the number of infection events at 12 and 24 months was 293 (31.9%: CI: 28.6–34.6) and 310 (35.3%, CI: 31.8–39.2), respectively. None of the infection events were significantly different from the placebo group. The results of the study by Team (2002) showed that after 6 weeks, HIV transmission rates were as follows: 5.7% (relative risk: 0.37; 95% CI: 0.21–0.65) for the longest zidovudine and lamivudine regimen, 8.9% (relative risk: 0.58; 95% CI: 0.36–0.94) for the intermediate zidovudine and lamivudine regimen, 14.2 (relative risk: 0.93; 95% CI: 0.62–1.40) for the shortest zidovudine and lamivudine regimen, and 15.3% for the placebo. The relative risk for the long and intermediate regimens was significantly lower than for the placebo.

### Adverse effects

Of the seven studies, four provided information on adverse effects. Drake *et al*.^28^ reported no differences in adverse events between the two groups. In the study by Nagot *et al*.^7^, the most common severe adverse events were anemia (n = 214; 17%), neutropenia (n = 138; 11%), malaria (24; 2%), hyponatremia (n = 22; 2%), and pneumonia (n = 20; 2%). Coovadia *et al*.^8^ reported that a total of 1,259 (83%) infants had adverse events, including gastroenteritis in 81 (5%) infants, pneumonia or bronchopneumonia in 73 (5%) infants, malaria in 54 (4%) infants, and kwashiorkor in 12 (1%) infants, with no significant difference between study groups. In the study by Fowler *et al*.^29^, clinical adverse events were common, with 83% of infants in either arm having at least one adverse event by 18 months (Table 1).

## DISCUSSION

The primary aim of this systematic review and meta-analysis was to investigate different interventions aimed at preventing mother-to-child transmission of HIV during breastfeeding. The study specifically analyzed randomized controlled trials (RCTs) evaluating interventions such as zidovudine regimens (with or without other antiretroviral therapies), zidovudine prophylaxis, zidovudine-lamivudine regimens, nevirapine and lopinavir-ritonavir regimens, valacyclovir regimens, and vitamin A supplementation. The goal was to assess their effectiveness in reducing mother-to-child transmission of HIV during breastfeeding.

The key findings of this study show that antiretroviral therapy (ART) significantly reduced the risk of HIV transmission during breastfeeding compared with placebo. According to the World Health Organization, mothers infected with HIV should receive lifelong antiretroviral therapy or antiretroviral prevention interventions to minimize HIV transmission during breastfeeding^31^. Similarly, the US Department of Health recommends a six-week course of zidovudine and/or three doses of nevirapine for infants within 48 hours of birth, with subsequent doses at specified intervals^22^. The use of antiviral drugs during pregnancy to reduce mother-to-child transmission has been shown to be more effective when combined with exclusive breastfeeding^32^. As emphasized by Neveu *et al*.^33^, combination antiretroviral therapy can reduce postpartum transmission of HIV to exclusively breastfed infants. These findings underscore the importance of ART interventions during breastfeeding to reduce the risk of transmission. In conclusion, the findings of the current meta-analysis are consistent with established recommendations, indicating that the use of zidovudine, nevirapine, and valacyclovir can decrease the risk of HIV transmission during breastfeeding^8,28-30^.

There is no difference in transmission between different types of ART interventions, and all these regimens can reduce the transmission rate. However, some interventions have a more pronounced effect in the short term and their effectiveness diminishes over time. The Petra Study Team highlighted that while zidovudine plus lamivudine regimens were effective in reducing HIV-1 transmission at six weeks postpartum, the benefits were significantly reduced after 18 months of follow-up^12^. Some regimens seem to be more effective in preventing transmission at low viral loads. In their study, Jamieson *et al.* ^30^ noted that the significant effect of zidovudine was observed specifically in women with low viral loads. Therefore, researchers emphasize the need to identify more effective and appropriate regimens for individuals with higher viral loads, even though ZDV has proven to be effective in preventing significant transmission risks.

However, despite efforts and the prescription of antiretroviral therapy for lactating mothers, the risk of HIV transmission is not completely eliminated^34^. To minimize the risk of HIV transmission during breastfeeding, it is generally recommended to maintain a plasma viral load below 100 copies/mL^35^. However, the presence of HIV RNA in breast milk, even at very low levels, highlights the challenges of achieving complete non-transmissibility^22^. Cases of mother-to-child transmission have been reported even with maternal plasma viral loads below 100 copies/mL^36^. There is a theoretical risk of transmission from infected epithelial cells in breast milk, even in mothers receiving antiretroviral therapy^37^. Therefore, there is no precise viral threshold that guarantees non-transmissibility^38^. As a result, preventing mother-to-child transmission of HIV during breastfeeding remains a complex and multifaceted challenge. Given this complexity, ongoing research is essential to improve strategies, ensuring the safety of breastfeeding while minimizing the risk of transmission.

This study suggests that the effectiveness of other treatments, such as high-dose vitamin A supplementation, remains unclear. The results of the study by Humphrey *et al.* ^11^ indicate that vitamin A supplementation, administered as a single high dose to either the mother or infant after delivery, had no significant effect on mother-to-child transmission during breastfeeding^11^. Overall, the evidence from all studies to date suggests that maternal vitamin A supplementation does not reduce postpartum mother-to-child transmission, regardless of dietary intake^39-41^. Consequently, the decision to implement postnatal maternal and newborn vitamin A supplementation programs in HIV-endemic areas should be based on an overall assessment of benefits, risks, and costs.

The findings support a growing body of evidence indicating that vitamin A supplementation may prolong survival in infants and children with HIV and should be considered in their care^11^. However, based on the available evidence, implementation of this form of supplementation may not be justified in terms of costs and risks in HIV-endemic areas^42-44^. Therefore, additional studies are needed to assess the effectiveness of vitamin A in preventing mother-to-child transmission of HIV and related complications.

Another notable finding from the review of RCTs was the occurrence of various complications in infants. These complications included anemia, neutropenia, malaria, hyponatremia, pneumonia, gastroenteritis, bronchopneumonia, and kwashiorkor. However, an important finding was that there was no significant difference in the incidence of complications between the antiretroviral therapy and placebo groups^7,8,29^. This result is important for understanding the safety profile of antiretroviral therapy compared with placebo, suggesting that the interventions studied may not significantly increase the risk of these complications in infants. The lack of significant differences between the study groups raises questions about the potential impact of the interventions on infant health, indicating that the observed complications may be due to factors other than the interventions. In addition, the results indicate that antiretroviral therapy, while effective in achieving its primary purpose, does not increase the susceptibility of infants to the mentioned complications compared with placebo. However, it is important to interpret these results with caution. The size of the study population and the specific characteristics of the trials may influence the generalizability of the findings. Therefore, further research and consideration of background factors are needed to confirm and generalize these results.

Studies investigating interventions to prevent mother-to-child transmission of HIV during breastfeeding have several advantages. This review examined a variety of interventions, including different antiretroviral therapy regimens and vitamin supplements, providing a comprehensive understanding of different approaches. The inclusion of a meta-analysis of four studies allowed for a pooled estimate of transmission risk, increasing statistical power and generalizability. Consistent findings across interventions showed homogeneity and increased confidence in their effectiveness. The reporting of adverse events in the studies is essential for evaluating the safety profile and potential risks associated with the interventions.

However, it is important to acknowledge the limitations of these studies. The different follow-up periods in the studies make it challenging to compare long-term effects. Some studies lacked placebo groups or included multiple study groups, making it difficult to identify specific intervention effects. In addition, the inclusion of different types of interventions in the selected studies may limit our ability to make direct comparisons and draw general conclusions about specific preventive measures. Overall, these findings provide valuable insights, but further research is needed to address these limitations and improve our understanding of the effectiveness and safety of these interventions.

## CONCLUSION

The findings of this study indicate that prescribing ART can effectively reduce the risk of mother-to-child transmission of HIV during breastfeeding. However, the effectiveness of certain interventions, such as high-dose vitamin A, requires further investigation. Clinicians and health care providers should consider the available evidence, individual patient factors, and local guidelines when making decisions about the prevention of mother-to-child transmission of HIV during breastfeeding. Additional research, including larger and more diverse studies, is essential to provide more definitive results and to guide clinical practice.
