# Supplementary material for: Interventions to prevent mother-to-child transmission in breastfeeding mothers with HIV: a systematic review and meta-analysis of randomized controlled trials
Source: Rev Inst Med Trop Sao Paulo. 2024 Jul 29;66:e45. doi: 10.1590/S1678-9946202466045 (PMC11295290; doi:10.1590/S1678-9946202466045)
Supplement: Supplementary file 1 [file 1678-9946-rimtsp-66-S1678-9946202466045-s1.pdf]

**Supplementary Material of the article "Interventions to prevent mother-to-child transmission in breastfeeding mothers with HIV: a systematic review and meta-analysis of randomized controlled trials"**

**Corresponding author:** Yun Wang
Jiangxi Maternal and Child Health Hospital, Obstetrical Department, Jiangxi, Nanchang, China
E-mail: wy13879132233@sina.com

**Title:** Search strategy

**Date of data collection:**
October 24, 2023

**Overview of data and files:**
This supplementary material is about the details of search strategies in different database including Web of Science, Scopus, PubMed, EMBASE, Cochrane Central Register of Controlled Trials, and Google Scholar. All these search strategies were piloting during October 2023 and finalized and run at October 24, 2023.

**Description of the methods of data collection or generation:**
First and the corresponding author in collaboration with an expert in data searching developed, piloted, and finalized the search strategies.

**Description of the methods used for data processing:**
All articles which were retrieved, entered and managed by Endnote reference manager program version 8.
