# Supplementary material for: Interventions to prevent mother-to-child transmission in breastfeeding mothers with HIV: a systematic review and meta-analysis of randomized controlled trials
Source: Rev Inst Med Trop Sao Paulo. 2024 Jul 29;66:e45. doi: 10.1590/S1678-9946202466045 (PMC11295290; doi:10.1590/S1678-9946202466045)
Supplement: Supplementary file 2 [file 1678-9946-rimtsp-66-S1678-9946202466045-s2.pdf]

REVISTA DO INSTITUTO DE MEDICINA TROPICAL DE SÃO PAULO

JOURNAL OF THE SÃO PAULO INSTITUTE OF TROPICAL MEDICINE

https://doi.org/10.48331/scielodata.Q7RKLS

Interventions to prevent mother-to-child transmission in breastfeeding mothers with HIV: a systematic review and meta-analysis of randomized controlled trials

Fangping Xu[1], Ying Xiong[1], Min Gu[1], Lingling Wan[1], Yun Wang[1]

[1]Jiangxi Maternal and Child Health Hospital, Obstetrical Department, Jiangxi, Nanchang, China

Correspondence to: Yun Wang
Jiangxi Maternal and Child Health Hospital, Obstetrical Department, Jiangxi, Nanchang, 330006, China

E-mail: wy13879132233@sina.com

Received: 28 February 2024

Accepted: 23 May 2024

SUPPLEMENTARY FILE S1

We developed search strategies tailored to each database search engine to optimize the relevance and precision of our results. Initially, we meticulously selected keywords based on a comprehensive literature review and MESH terms. However, due to variations in search engine algorithms, some databases necessitated adjustments in our search strategy. For instance, while one database accepted wildcard characters like "*" at the beginning of a keyword, others only permitted them at the end. As a result, we customized our approach accordingly to optimize search results across all platforms.

Web of Science (filters: English, article, proceeding paper, early access)**:**

*(TS=(breastf*d*) OR TS=("breast f*d*") OR TS=("breast milk")) AND (TS=(Antepart*) OR TS=(*natal) OR TS=(*partum) OR TS=(puerper*)) AND (TS=(HIV*) OR TS=("human immunodeficiency virus*") OR TS=(AIDS) OR TS=("Acquired Immune Deficiency Syndrome") OR TS=(*HTLV-III) OR TS=("Human T Cell Lymphotropic Virus Type III") OR TS=("Human T-Cell Leukemia Virus Type III")) AND (TS=(Infect*) OR TS=(Transmi*) OR TS=(Vertical) OR TS=("Maternal-Fetal") OR TS=("Mother to Child") OR TS=(Fetomaternal))*

Scopus (filters: English, article)**:**

*(TITLE-ABS(breastf*d*) OR TITLE-ABS("breast f*d*") OR TITLE-ABS("breast milk")) AND (TITLE-ABS(Antepart*) OR TITLE-ABS(*natal) OR TITLE-ABS(*partum) OR TITLE-ABS(puerper*)) AND (TITLE-ABS(HIV*) OR TITLE-ABS("human immunodeficiency virus*") OR TITLE-ABS(AIDS) OR TITLE-ABS("Acquired Immune Deficiency Syndrome") OR TITLE-ABS(*HTLV-III) OR TITLE-ABS("Human T Cell Lymphotropic Virus Type III") OR TITLE-ABS("Human T-Cell Leukemia Virus Type III")) AND (TITLE-ABS(Infect*) OR TITLE-ABS(Transmi*) OR TITLE-ABS(Vertical) OR TITLE-ABS("Maternal-Fetal") OR TITLE-ABS("Mother to Child") OR TITLE-ABS(Fetomaternal))*

PubMed (filters*: None)**:**

(breastfeed[Title/Abstract] OR breastfed[Title/Abstract] OR "breast feed*"[Title/Abstract] OR "breast fed"[Title/Abstract] OR "breast milk"[Title/Abstract]) AND (antepart*[Title/Abstract] OR postnatal[Title/Abstract] OR antenatal[Title/Abstract] OR prenatal[Title/Abstract] OR perinatal[Title/Abstract] OR antepart*[Title/Abstract] OR postpart*[Title/Abstract] OR puerper*[Title/Abstract]) AND (HIV[Title/Abstract] OR HIV-1[Title/Abstract] OR HIV-2[Title/Abstract] OR "human immunodeficiency virus*"[Title/Abstract] OR AIDS[Title/Abstract] OR "Acquired Immune Deficiency Syndrome"[Title/Abstract] OR HTLV-III[Title/Abstract] OR LAV-HTLV-III[Title/Abstract] OR HTLV-III/LAV[Title/*

This is an open-access article distributed under the terms of the Creative Commons Attribution License.

Abstract] OR "Human T Cell Lymphotropic Virus Type III"[Title/Abstract] OR "Human T-Cell Leukemia Virus Type III"[Title/Abstract]) AND (Infect[Title/Abstract] OR Transmi*[Title/Abstract] OR Vertical[Title/Abstract] OR "Maternal-Fetal"[Title/Abstract] OR "Mother to Child"[Title/Abstract] OR Fetomaternal[Title/Abstract])*

EMBASE (filters*: English, article, article in press, preprint, humans)**:**

*(breastf*d*:ab,ti OR 'breast f*d*':ab,ti OR 'breast milk':ab,ti) AND (postnatal:ab,ti OR antenatal:ab,ti OR prenatal:ab,ti OR perinatal:ab,ti OR antepart*:ab,ti OR postpart*:ab,ti OR puerper*:ab,ti) AND (hiv*:ab,ti OR 'human immunodeficiency virus*':ab,ti OR aids:ab,ti OR 'acquired immune deficiency syndrome':ab,ti OR 'htlv iii*':ab,ti OR 'lav htlv iii':ab,ti OR 'human t cell lymphotropic virus type iii':ab,ti OR 'human t-cell leukemia virus type iii':ab,ti) AND (infect*:ab,ti OR transmi*:ab,ti OR vertical:ab,ti OR 'maternal-fetal':ab,ti OR 'mother to child':ab,ti OR fetomaternal:ab,ti)*

Cochrane Central Register of Controlled Trials (filters*: English, trials)**:**

((breastfeed):ti,ab,kw OR (breastfed):ti,ab,kw OR ("breast fed"):ti,ab,kw OR (breast NEXT feed*):ti,ab,kw OR ("breast milk"):ti,ab,kw) AND ((Antepart*):ti,ab,kw OR (*natal):ti,ab,kw OR (*partum):ti,ab,kw OR (puerper*):ti,ab,kw) AND ((HIV*):ti,ab,kw OR ("human immunodeficiency virus"):ti,ab,kw OR (AIDS):ti,ab,kw OR ("Acquired Immune Deficiency Syndrome"):ti,ab,kw OR (*HTLV-III):ti,ab,kw OR ("Human T Cell Lymphotropic Virus Type III"):ti,ab,kw OR ("Human T-Cell Leukemia Virus Type III"):ti,ab,kw) AND ((Infect*):ti,ab,kw OR (Transmi*):ti,ab,kw OR (Vertical):ti,ab,kw OR ("Maternal-Fetal"):ti,ab,kw OR ("Mother to Child"):ti,ab,kw OR (Fetomaternal):ti,ab,kw)*

Google Scholar (filters*: None)**:**

allintitle: breastfeeding HIV transmission

* After retrieving search results, filters were applied using the filter panel on the results page.
